# Cellular Responses to Extracellular Vesicles as Potential Markers of Colorectal Cancer Progression

**DOI:** 10.3390/ijms242316755

**Published:** 2023-11-25

**Authors:** Sonia Guarnerio, Robert Tempest, Rawan Maani, Stuart Hunt, Laura M. Cole, Christine L. Le Maitre, Keith Chapple, Nicholas Peake

**Affiliations:** 1 Biomolecular Sciences Research Centre, Sheffield Hallam University, Sheffield S1 1WB, UK; sonia.guarnerio@newcastle.ac.uk (S.G.); r.maani@shu.ac.uk (R.M.);; 2 NanoFCM Co., Ltd., Medicity, Nottingham NG90 6BH, UK; 3 School of Clinical Dentistry, University of Sheffield, Sheffield S10 2TN, UK; s.hunt@sheffield.ac.uk; 4 Division of Clinical Medicine, University of Sheffield, Sheffield S10 2TN, UK; c.lemaitre@sheffield.ac.uk; 5 Colorectal Surgical Unit, Sheffield Teaching Hospitals NHS Foundation Trust, Sheffield S10 2JF, UK; keith.chapple@nhs.net

**Keywords:** colorectal cancer, extracellular vesicles, responsive biomarkers

## Abstract

The development of novel screening tests aims to support early asymptomatic diagnosis and subtyping patients according to similar traits in the heterogeneous cancer cohort. Extracellular vesicles (EVs) are promising candidates for the detection of disease markers from bodily fluids, but limitations in the standardisation of isolation methods and the intrinsic EV heterogeneity obtained from liquid biopsies are currently obstacles to clinical adoption. Here, cellular responses to cancer EVs were initially explored as potential complementary biomarkers for stage separation using colorectal cancer (CRC) SW480 and SW620 cell line models. A pilot study on a small cohort of CRC patients and controls was then developed by performing a multivariate analysis of cellular responses to plasma-derived EVs. Several cell activities and markers involved in tumour microenvironment pathways were influenced by the treatment of cell line EVs in a stage-dependent manner. The multivariate analysis combining plasma EV markers and cellular responses to plasma EVs was able to separate patients according to disease stage. This preliminary study offers the potential of considering cellular responses to EVs in combination with EV biomarkers in the development of screening methods.

## 1. Introduction

Colorectal cancer (CRC) or bowel cancer is the third most common cancer worldwide, being responsible for 10% of cancer-related deaths in developed countries [1,2]. Standard diagnostic procedures, such as colonoscopy, are invasive, require prior bowel preparation, and can be uncomfortable. In addition, the procedure carries small but significant risks and has an associated financial burden [3]. However, the diagnosis of more CRC patients at an early stage would greatly improve prognosis since the survival rate at stage 1 is about 90%, which decreases to less than 10% at stage 4 [4].

Alongside the development of new therapeutics, non-invasive modalities of screening CRC have helped in reducing the levels of patient death in the past decades [5,6,7]. The long progression of CRC offers a promising window for the development of screening tests, from the long time required for the formation of polyps to the asymptomatic transition from early to late stages [7]. Two screening tests based on faecal samples have been introduced to support early detection of CRC, but there is a need to improve screening strategies, as they often lack in sensitivity and specificity [7]. Liquid biopsies, such as blood, urine, and saliva, could provide an additional source of disease markers, which represent easily measured detection alternatives to traditional invasive approaches. By combining promising molecular targets, it may be possible to develop a highly sensitive and specific screening approach, which can also determine known variations of the disease and help support personalised therapy.

Extracellular vesicles (EVs) are vesicles enclosed by a bilayer lipidic membrane and secreted by all cell types, including cancer cells. They can travel in all biofluids and contain bioactive cargo defined by the donor cell. Colorectal cancer cell lines have extensively been profiled for their cargo content, showing stage-dependent differences in proteomic and transcriptomic signatures [8,9]. In addition to their functional cargos, the double membrane confers stability during circulation, making them one of the most interesting approaches employed by researchers to identify new and improved biomarkers in liquid biopsies [10,11]. Despite their potential, there are some limitations in using EVs as biomarkers. Circulating EVs found in different biological fluids can be strongly heterogeneous in phenotype and cargo. In a study on lung cancer, differences in the proteomes contained in EVs isolated from saliva and EVs isolated from serum were identified [12]. Thus, the liquid biopsy needs to be chosen carefully. Furthermore, information derived from EVs can be strongly dependent on the method of isolation chosen [13,14]. Furthermore, the yield of cancer-related biomarkers contained in a heterogeneous population of circulating EVs is generally low and thus hard to determine. This requires sensitive techniques that are generally expensive but also time consuming, and are therefore incompatible with clinical procedures and timelines. The potential lack of robustness and the heterogeneity of circulating EVs do pose serious limitations, slowing the process of introducing them into clinical workflows for diagnosis. Recently, a promising match of membrane proteins present on EVs (CD147 and A33) have been shown to distinguish between CRC patients and controls, but similar limitations were discussed [15].

These limitations may become less influential if EV bioactivities, rather than EV profiling, are considered. Extensive research around functional EV roles could help in identifying specific cellular responses working as cancer biomarkers. For example, it was shown that blood EVs in women affected by breast cancer could induce angiogenesis in HUVEC cells compared to blood EVs from healthy women [16]. In CRC, EVs have been associated with numerous processes, from serving the cellular crosstalk in the tumour microenvironment to the formation of the pre-metastatic niche [17]. EVs isolated from CRC cells can induce phenotypical reprogramming of normal colonic cells towards aggressive behaviours [18], and this was also observed in plasma EVs obtained from CRC patients [19]. The focus on cellular responses could reduce the need to improve pipeline sensitivity from EV purification to marker analysis. However, a singular cellular response is not likely to be specific and reliable enough to be implemented in alternative screening techniques since cancer progression is associated with several “hallmarks,” or processes, rather than a single feature. Due to enriched and specialised EV cargos, specific EV types generally induce more than one effect in cells, thus providing an interesting new perspective in the research of EV-related biomarkers for both diagnosis and prognosis.

Here, a screening approach based on the evaluation of multiple cellular responses to CRC-derived EVs was investigated. This study tested the hypothesis that cellular responses to CRC-derived EVs can determine the stage of the disease. First, EVs from SW480 (primary stage) and isotypic SW620 (metastatic stage) cell lines were used to treat SW480 cancer cells and MRC5 fibroblasts to determine a panel of potential biomarkers, which were then applied ex vivo with patient plasma-derived EVs. Finally, multivariate analysis was developed to evaluate the strength of the preliminary screening model.

## 2. Results

### 2.1. Isolation and Characterisation of SW480 and SW620 EVs

**Figure 1:**
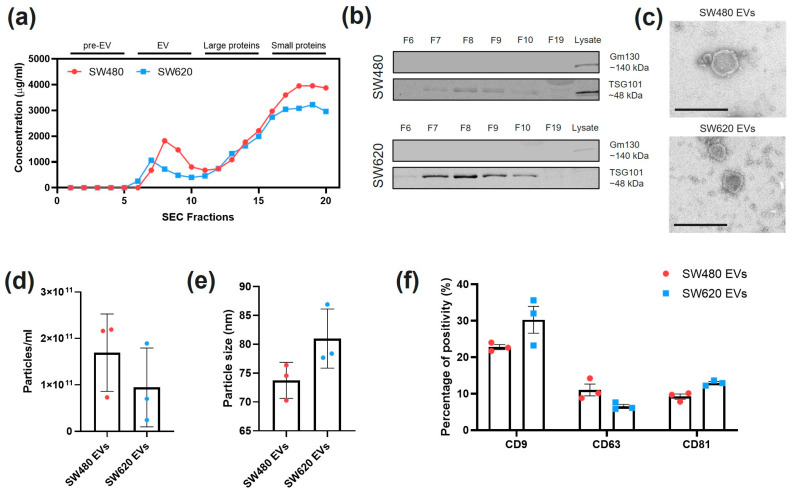

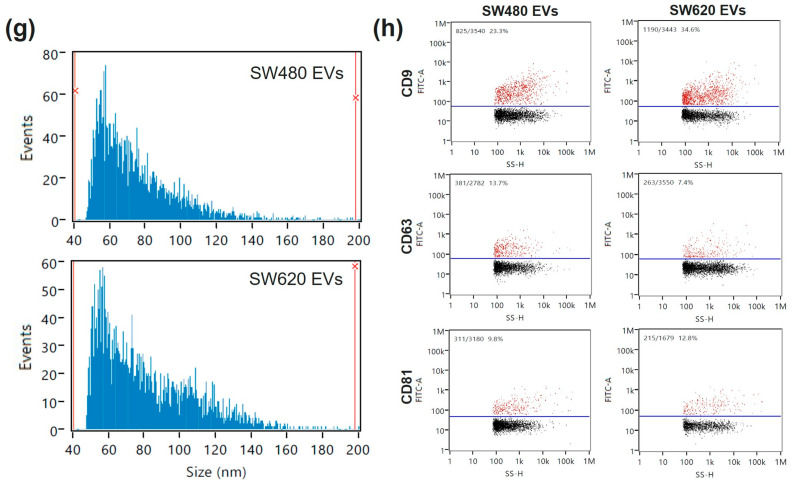
Characterisation of SW480 and SW620 EVs. (**a**) BCA assay of the SEC fractions collected from SW480 and SW620 cell lines. (**b**) Western blots for GM130 (non-EV marker) and TSG101 of SEC fractions. Cell lysates from SW480 and SW620 as control. (**c**) Representative TEM images of SW480 and SW620 EVs. Scale bar = 100 nm. (**d**) Particle concentrations and (**e**) particle sizes of SW480 and SW620 EVs obtained with nanoflow cytometry. (**f**) Percentage of positivity for EV tetraspanin markers CD9, CD63, and CD81 detected by nanoflow cytometry after immunolabelling SW480- and SW620-derived EVs. (**g**) Representative nanoflow cytometry histograms showing event/size. Red lines and x defines the area for analysis. (**h**) Representative nanoflow cytometry scatter plots of tetraspanin-positive particle events in SW480 and SW620 EVs. Blue lines define the gate for positivity.

Fractions collected in SEC were quantified for protein content by the BCA assay, which showed two distinct peaks (Figure 1a): a first peak at fractions 5–10 and a second peak of soluble proteins that reached saturation at fraction 18. The earlier peak was assumed to contain EVs due to its size, and protein concentrations ranged between 500 and 2000 µg/mL, with SW480 elution generally higher compared to SW620 elution. To confirm the presence of EVs in the SEC fractions, western blotting for EV marker TSG101 and non-EV marker GM130 was performed (Figure 1b). TSG101 was found to be expressed in SW480 fractions 7–10, SW620 fractions 7–10, and in the SW480 lysate. On the contrary, GM130 was found to be expressed exclusively in the cell lysates. The three fractions with highest concentrations in this EV-enriched peak were then pooled and the presence of EVs was confirmed by visualising the morphology by TEM (Figure 1c). The particle concentration of the pooled fractions was around 10^11^ particles/mL for both SW480 and SW620 EVs (Figure 1d). SW480 EVs were not significantly smaller in average size than SW620 EVs (*p* = 0.1), although both EVs showed a size distribution with the peak diameter at ~60 nm (Figure 1e,g). Tetraspanin markers CD9, CD63, and CD81 were then labelled and quantified by nanoflow cytometry (Figure 1f,h). SW480 and SW620 EVs were mainly positive for CD9, with 20% positivity for SW480 EVs and 30% positivity for SW620 EVs. Positivity for both CD63 and CD81 was ~10% for both SW480 and SW620 EVs.

### 2.2. Cellular Activity Is Influenced by SW480 and SW620 EVs

**Figure 2:**
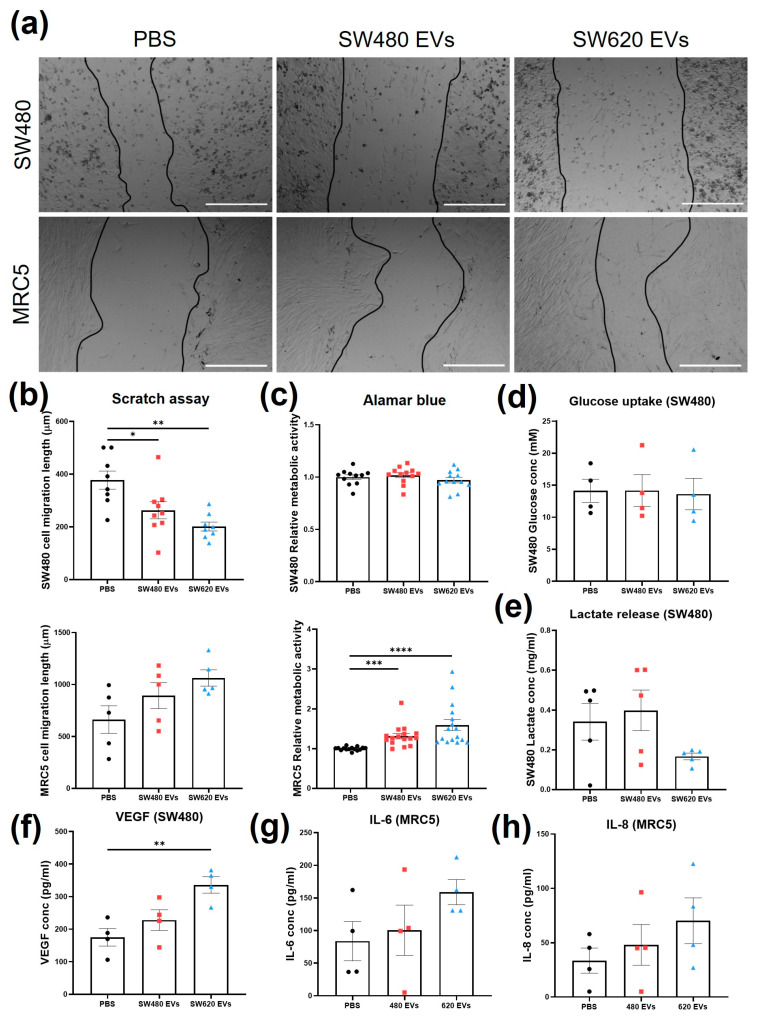
Cellular responses upon treatment with SW480 EV, SW620 EV, or PBS. (**a**) Representative images of scratch migration assay of SW480 and MRC5 cells at 36 h of treatment. Scale bar = 500 µm. (**b**) Migration length of SW480 and MRC5 cells at 36 h of treatment. (**c**) SW480 and MRC5 cell metabolic activity with Alamar blue assay. (**d**) Glucose uptake of SW480 cells. (**e**) Lactate production by SW480 cells. (**f**) VEGF concentration of SW480 cells. (**g**) IL-6 release by MRC5 cells. (**h**) IL-8 release by MRC5 cells. All data are presented as mean ± SEM; n ≥ 3 per group. One-way ANOVA with Tukey’s post hoc test, * *p* < 0.05, ** *p* < 0.01, *** *p* < 0.001, **** *p* < 0.0001.

To establish potential bioactivity markers, migration, cell viability, and metabolic activity of SW480 and MRC5 cells were evaluated after treatment with 50 µg/mL (protein content) SW480 and SW620 EVs. The concentration was chosen according to previous literature [20]. Cell migration was assessed by scratch assay at four time points: 0, 8, 24, and 36 h (Figure S1, Supplementary Material). Length measurements were taken from the borders of cells still adhering, as single cell migration was undistinguishable from detached cells or cell death. At 36 h, both CRC EVs induced statistically significant decreases in the migration of SW480 cells compared to the PBS control (Figure 2a,b). The effect was higher with SW620 EVs (*p* < 0.01) compared to SW480 EVs (*p* < 0.05) (Figure 2b). Both SW480 and SW620 EVs increased MRC5 cell migratory ability, but the increases were not statistically significant (Figure S1, Supplementary Material). At 36 h, a higher increase was linked to SW620 EVs (*p* = 0.0707) compared to SW480 Evs (*p* = 0.3577) (Figure 2b). SW480 cell metabolic activity was not affected by treatment with CRC Evs, as indicated by the Alamar blue assay (Figure 2c). MRC5 cells treated with both CRC Evs showed statistically significant increases in metabolic activity compared to the control (*p* < 0.001 SW480 Evs, *p* < 0.0001 SW620 Evs, Figure 2c). CRC Evs did not affect the glucose uptake of the cells, whereas SW620 Evs decreased the production of lactic acid by SW480 cells (*p* > 0.05) (Figure 2d,e). The level of VEGF-165 (VEGF-A) released by SW480 cells was significantly increased after SW620 EV treatment compared to the control (*p* < 0.05) (Figure 2f). IL-6 release was upregulated when MRC5 cells were treated with SW620 Evs compared to the control (*p* = 0.2382) and SW480 Evs (*p* = 0.3972), but the increase was not statistically significant (Figure 2g). SW480 EV treatment also resulted in a small increase in IL-6 release (not statistically significant, *p* = 0.9204). Similarly, IL-8 release levels were higher in cells treated with SW480 Evs (*p* = 0.8331), and even more after SW620 EV conditioning, although no statistical significance was observed (*p* = 0.3447, Figure 2h).

### 2.3. Identification of Novel Secretory Targets Regulated by CRC Evs

**Figure 3:**
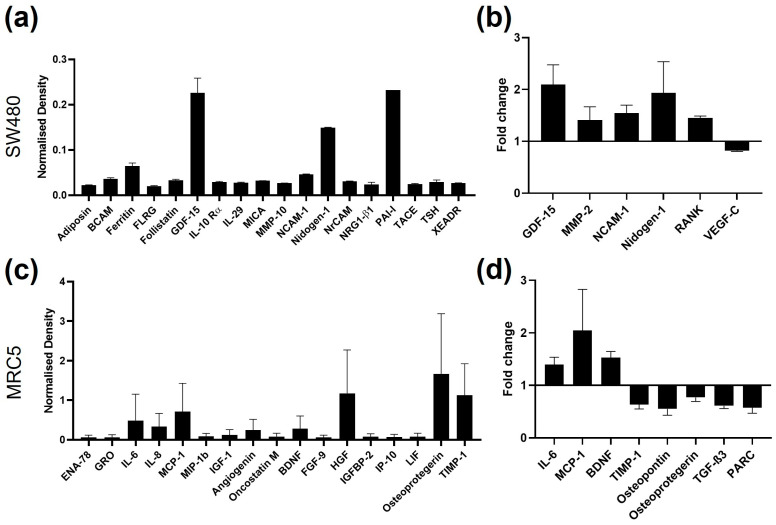
Cytokine array signals of supernatants from SW480 and MRC5 cells. (**a**) SW480 cytokine signals with density found in the PBS control samples higher than 0.01; n = 2. (**b**) SW480 cytokine signals with fold change between PBS control and SW620 EV-treated samples constant between biological replicates; n = 2. (**c**) MRC5 cell cytokine signals with density of the PBS control samples higher than 0.01; n = 2. (**d**) MRC5 cell cytokine signals with fold change between PBS control and SW620 EV-treated samples constant between the biological replicates; n = 2.

To expand the panel of potential measures that could show responses to CRC EVs, the supernatants from SW480 cells treated with SW620 EVs were compared to those of untreated cells using a cytokine array of 51 targets (including proteolytic enzymes) and the full list of results can be found in Table S1, Supplementary Material. The threshold of signal intensity >0.01 was reached by 18 cytokines (Figure 3a). Cytokines with consistent fold change (SW620Evs/PBS) among biological replicates were considered for the selection of the most differentially expressed markers (Figure 3b), resulting in six cytokines: GDF-15, MMP-2, NCAM-1, Nidogen-1, RANK, and VEGF-C. Similarly, a cytokine array containing 80 immune-related cytokines was undertaken on the supernatants from MRC5 fibroblasts treated with SW620 EVs and compared to those of untreated fibroblasts (Table S2, Supplementary Material). Seventeen cytokines were found to be highly expressed (>0.01) by MRC5 fibroblasts (Figure 3c). When the difference between control and treatment was considered, 8 cytokines (IL-6, MCP-1, BDNF, TIMP-1, osteopontin, osteoprotegerin, TGF-β3, and PARC) were found with constant fold change between biological replicates (Figure 3d).

### 2.4. Plasma EV Characterisation and Tetraspanin Levels in Plasma

**Figure 4:**
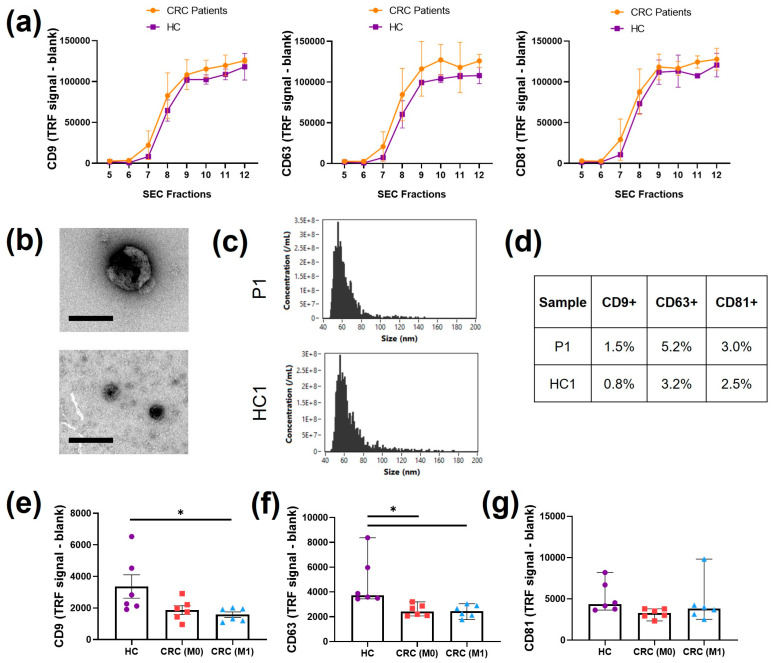
Plasma EV characterization. (**a**) DELFIA immunosensitive assay for tetraspanins CD9, CD63, and CD81 in isolated SEC fractions; n = 3. (**b**) Representative TEM images of different sizes of Evs found in the pooled SEC fractions from CRC patients. Scale bar = 100 nm. (**c**) Representative nanoflow cytometry event/size curves of CRC patients and HCs. (**d**) Percentage of positivity of the three tetraspanin markers, CD9, CD63, and CD81, detected with nanoflow cytometry of CRC patients and HCs. (**e**) DELFIA immunosensitive assay for CD9 found in pre-SEC plasma derived from HCs and CRC patients (M0, M1); n = 6, mean ± SEM, one way ANOVA, * *p* < 0.05. (**f**) DELFIA immunosensitive assay for CD63 found in pre-SEC plasma derived from HCs and CRC patients (M0, M1); n = 6, median ± 95% CI, Kruskal–Wallis * *p* < 0.05. (**g**) DELFIA immunosensitive assay for CD81 found in pre-SEC plasma derived from HCs and CRC patients (M0, M1); n = 6, median ± 95% CI, Kruskal–Wallis.

Isolation of plasma Evs was conducted by SEC to maintain consistency with cell line isolation. Since the levels of markers in plasma Evs are generally difficult to detect, a high-sensitivity immune-based approach through DELFIA was employed to screen for CD9, CD63, and CD81 in SEC fractions 5–12 (Figure 4a). The markers were expressed in fractions 8, 9, 10, 11, and 12, but expression was absent or low from fractions 5 to 7. For this reason, a new pool of Evs (8–11) was used and characterised. The morphology of the pooled Evs was visualised with TEM (Figure 4a). Heterogeneous sizes of particles showing bilayer membranes were found from 50 nm to 100 nm. The event/size curve from nanoflow cytometry analysis showed a similar profile to the cell line curves, with the main peak at 60 nm (Figure 4b). After immunolabelling, the percentage of positivity with nanoflow cytometry for CD9, CD63, and CD81 was found to be lower than 10% for all three tetraspanins (Figure 4c). The highest positivity was found for CD63, which was above 3% in Evs from both CRC patients and HCs.

The DELFIA immunoassay was also used to evaluate and compare the detection of CD9, CD63, and CD81 on pre-SEC plasma derived from the three groups of patients chosen (HC, M0, and M1) as a first screening of differences between the patients (Figure 4e–g). All three tetraspanins were found with detectable levels, but lower than the values in individual SEC fractions. The CD9 level was lower in M0 plasma (*p* = 0.0932) and significantly lower in M1 plasma compared to that in HC plasma (*p* < 0.05, Figure 4e). Similarly, a statistically significant decrease in the CD63 level was observed in both M0 and M1 patient plasma (*p* < 0.05, Figure 4f). No main differences were observed in the levels of CD81 (Figure 4g).

### 2.5. Plasma EVs Influence Cancer Hallmarks

**Figure 5:**
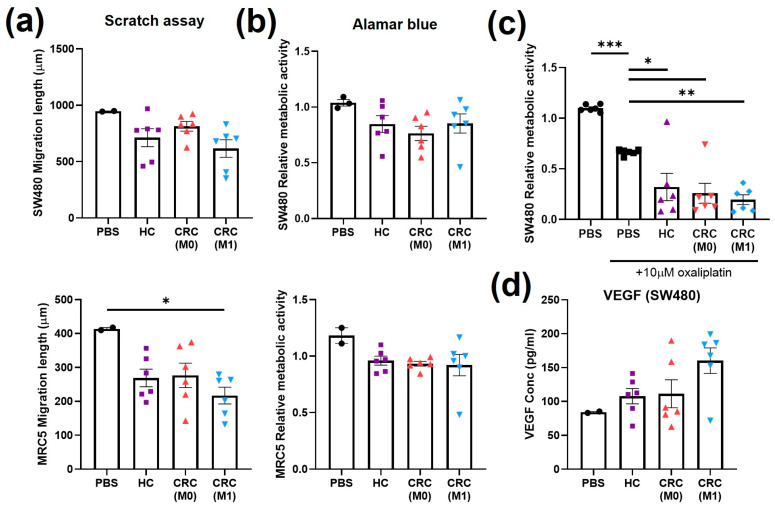
Cell migration, cell metabolism, drug resistance, and angiogenesis after treatment with plasma EVs. (**a**) Scratch assay of SW480 and MRC5 cells at 36 h of treatment with plasma EVs or PBS. (**b**) Alamar blue assay on SW480 and MRC5 cells after 24 h treatment with plasma EVs or PBS. (**c**) Alamar blue assay on SW480 cells after treatment with plasma EVs or PBS in combination with oxaliplatin. (**d**) VEGF levels in SW480 cell supernatant after treatment with plasma EVs or PBS. All data are presented as mean ± SEM; n = 6 per group. One-way ANOVA with Tukey’s post hoc test, * *p* < 0.05, ** *p* < 0.01, *** *p* < 0.001.

The assays previously performed to test the responses of cells to CRC EVs derived from cell lines were then applied to evaluate the responses to plasma EVs derived from patients. The assays were grouped according to their roles as cancer hallmarks. The treatments with plasma EVs were normalised to the volume, as no significant differences in particle concentration were found between patients (Figure 4c). An equal volume of PBS was also used as a control to normalise against the elution of the EV treatment. SW480 and MRC5 cell migration was generally reduced by conditioning with all plasma EV types compared to the PBS control (*p* > 0.05, Figure 5a) but was only significantly lower when MRC5 cells were treated with M1 plasma EVs (*p* < 0.05). A non-significant decrease (*p* > 0.05) in metabolic activity indicated by the Alamar blue assay after plasma EV conditioning compared to the PBS control was also observed, independent of the origin of the plasma (Figure 5b). The metabolic assays (glucose uptake and lactate production), which did not show significant differences after cell line EV conditioning, were replaced by analysis of SW480 drug resistance by co-treatment with plasma EVs and oxaliplatin. Significant decreases in metabolic activity were observed between the PBS control and oxaliplatin-only control (*p* < 0.001), oxaliplatin-only control and HC (*p* < 0.05), oxaliplatin-only control and M0 (*p* < 0.05), and oxaliplatin-only control and M1 (*p* < 0.01) (Figure 5c). M1 plasma EV conditioning induced non-significant increases in VEGF-A levels found in SW480 supernatant compared to the PBS control (*p* = 0.1509), HC plasma EVs (*p* = 0.1657), and M0 plasma EVs (*p* = 0.2114) (Figure 5d).

### 2.6. Plasma EVs Influence Emerging Cancer Hallmarks

**Figure 6:**
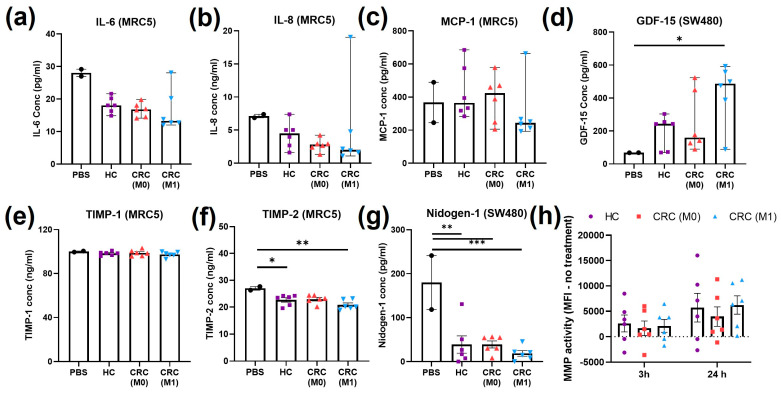
Inflammatory and matrix remodelling cell markers after treatment with plasma EVs. (**a**) IL-6 from MRC5 cell supernatant after treatment with plasma EVs or PBS; median ± 95% CI, Kruskal–Wallis. (**b**) IL-8 from MRC5 cell supernatant after treatment with plasma EVs or PBS; median ± 95% CI, Kruskal–Wallis. (**c**) MCP-1 from MRC5 cell supernatant after treatment with plasma EVs or PBS; median ± 95% CI, Kruskal–Wallis. (**d**) GDF-15 from SW480 cell supernatant after treatment with plasma EVs or PBS; median ± 95% CI, Kruskal–Wallis, * *p* < 0.05. (**e**) TIMP-1 from MRC5 cell supernatant after treatment with plasma EVs or PBS; mean ± SEM, one-way ANOVA. (**f**) TIMP-2 from MRC5 cell supernatant after treatment with plasma EVs or PBS; mean ± SEM, one-way ANOVA, * *p* < 0.05, ** *p* < 0.01. (**g**) Nidogen-1 from SW480 cell supernatant after treatment with plasma EVs or PBS; mean ± SEM, one-way ANOVA, ** *p* < 0.01, *** *p* < 0.001. (**h**) MMP activity found in SW480 cells at 3 h and 24 h of treatment with plasma EVs; mean ± SEM, one-way ANOVA.

Further analysis of inflammatory and matrix remodelling features recently defined in the hallmarks of cancer were performed after treatment with plasma EVs. Levels of IL-6 released by MRC5 cells decreased (not significantly, *p* > 0.05) with all plasma EV treatments compared to the PBS control (Figure 6a). Similarly, non-significant decreases (*p* > 0.05) in IL-8 levels in the MRC5 cell supernatant were observed after all plasma EV treatments compared to the PBS control (Figure 6b). One value in the M1 group was separated compared to rest of the patients in both IL-6 and IL-8. No main differences were found in MCP-1 from the MRC5 cell supernatant (Figure 6c). GDF-15 was tested in the SW480 supernatant and levels increased after plasma EV treatments, with a statistically significant increase after M1 plasma EV treatment compared to the PBS control (*p* < 0.05, Figure 6d). No main differences were found in TIMP-1 from the MRC5 cell supernatant (Figure 6e). TIMP-2 levels in MRC5 were significantly reduced after HC (*p* < 0.05) and M1 (*p* < 0.01) plasma EV treatments and non-significantly reduced after M0 plasma treatment (*p* = 0.0517) (Figure 6f). Nidogen-1 levels in the SW480 supernatant were significantly lower after all three plasma EV treatments compared to the PBS control (HC *p* < 0.01, M0 *p* < 0.01, and M1 *p* < 0.001) (Figure 6g). Finally, the matrix metalloproteinase activity of SW480 cells was tested at two different time points, but no significant changes were observed between the plasma EV treatment groups (Figure 6h).

### 2.7. Pilot Multivariate Analysis Discriminate CRC Patients on the Basis of Cellular Responses to Plasma EVs and Power Analysis

**Figure 7:**
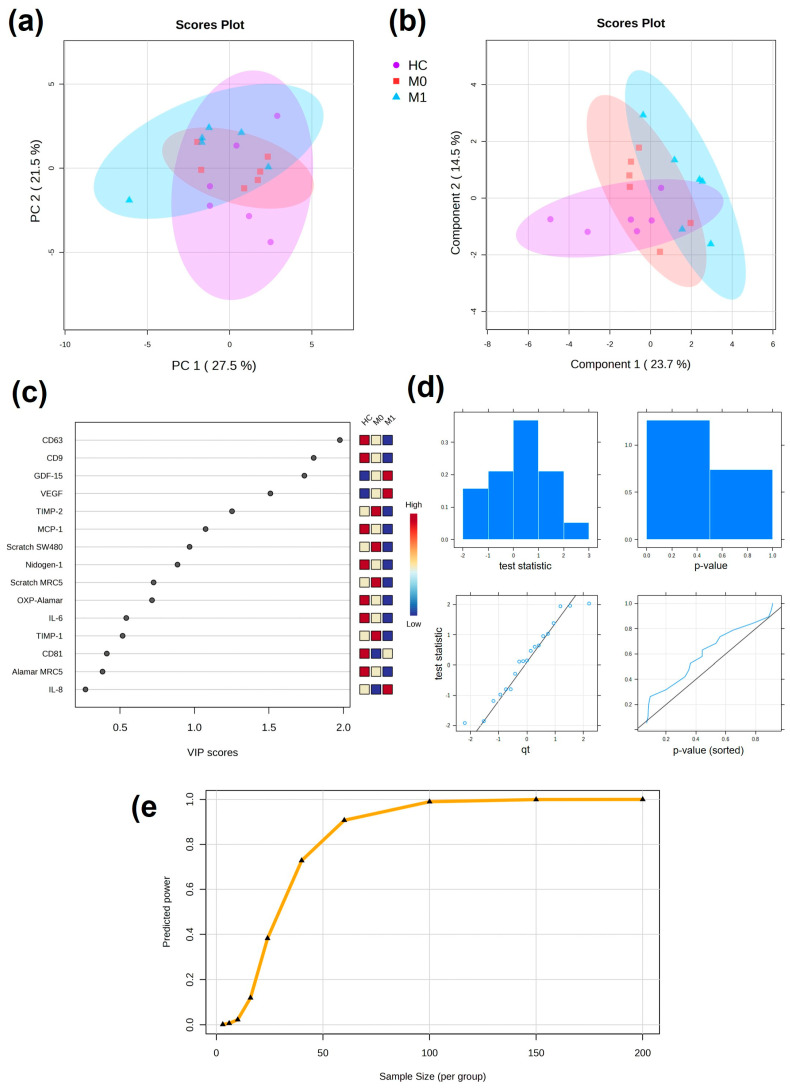
Multivariate analysis of cellular responses to plasma EVs. (**a**) Unsupervised PCA with 95% confidence ellipses. (**b**) Supervised PLS-DA with 95% confidence ellipses. (**c**) VIP scores from PLS-DA showing discriminating variables. Important features are found with VIP score > 1. (**d**) Plots overviewing the test statistics and *p*-values calculated from the pilot data to evaluate whether they follow a normal distribution. (**e**) Predicted power curve for FDR = 0.1 (significance criterion) and maximum size cohort of 100 patients per group. Curve saturation at 100 patients per group.

All of the results obtained from the cellular response assays (from both SW480 and MRC5 cells) for the evaluation of plasma EVs were next applied to a multivariate analysis model. Initially, PCA was performed as unsupervised analysis to investigate the ability to cluster patients together (Figure 7a). Partial separation was observed between the HC and M1 groups. When supervised PLS-DA was performed, which considered information about the patient groups, single values within groups were found in more defined clusters: the HC group was partially separated from M0 and M1 by both components, whereas the M0 and M1 groups were separated by component 1 (Figure 7b). From the PLS-DA, VIP scores can be obtained to evaluate variables that contribute more to group-dependent separation. Variables with a VIP score larger than 1 are most important for the separation. CD63, CD9, GDF-15, and VEGF had VIP scores over 1.5 (Figure 7c).

Power analysis was performed to obtain information about the size of the cohort based on the preliminary results on the cellular assays performed in the pilot study. Data were normally distributed and the predicted power curve was saturated at 100 patients per group (Figure 7d,e).

## 3. Discussion

EVs have great potential as circulating biomarkers due to their disease-relevant cargo and, as such, their translation into clinical use is currently being explored [11,21,22] and promising biomarkers expressed by EVs found in faecal samples have been recently identified [15]. However, the isolation procedures and the sensitivity of EV characterisation for disease-specific markers from liquid biopsy are challenging and cannot often be sustained in a patient-based study. Moreover, the selection of individual biomarkers often does not provide information about the stage of the disease. It was recently demonstrated that EVs originating from different sources can induce differential responses on recipient cells [23]. In this preliminary study, cellular responses to EVs were evaluated as alternative and supportive potential biomarkers of differential CRC stage using multiplexed cell-based bioassays.

Due to their isogenic origin, SW480 and SW620 cell lines offer an interesting in vitro model in which to observe differences in the effect of stage-dependent EVs [24,25]. Therefore, they were initially chosen as cell sources of EVs in the exploration of cellular and molecular responses in the TME. In most of the cellular and molecular assays tested, SW620 EVs induced more relevant changes than SW480 EVs, which confirmed a functional impact dependent on the highly mutated EV cargo.

Cancer cells adapt their metabolism to address the requirements of the continuously changing TME during the steps leading to metastasis [26]. The reduction of lactic acid production, an indicator of anaerobic glycolysis, after treatment with SW620 EVs might imply the metabolic change previously observed to be representative of phenotypic changes towards aggression in other types of cancer [27,28]. This was supported by the increase in levels of VEGF-A, a marker of angiogenesis, upon CRC EV treatment and was in line with previous studies [29]. The decrease in cancer cell migration quantified by the scratch assay upon EV treatment, which at first would seem to contradict the hypothesis, may not be an ideal measure of invasive/migratory behaviour as it lacked the ability to differentiate between migration and proliferation and was sensitive to methodological confounders such as cell debris, which prevented automated quantitation. Given that better invasive models are time-intensive and technically sensitive for possible routine use, it was concluded that secretory factors as surrogate markers of invasion in addition to other hallmarks may be better and more straightforward measurements in a clinical biomarker context.

The phenotypic switch from normal fibroblasts to CAFs has also been associated with cancer-derived EVs in different types of cancer [30,31]. A significant increase in proliferation and migration of MRC5 cells was induced by CRC EVs, supporting the idea that fibroblasts are activated by EV cargo [32]. Furthermore, higher expression of pro-inflammatory IL-6 after CRC EV treatment was observed. The interleukins released by CAFs have been previously found to be responsible for different aspects of cancer progression, from inducing EMT in gastric cancer to drug resistance in prostate cancer [33,34].

Novel secretory targets associated with the TME and cancer processes were identified through cytokine arrays tested on both cancer cells and fibroblasts, such as GDF-15, which is a member of the TGF-β family and has been extensively linked to metastasis in different cancers [35,36,37]; nidogen-1, which has also been previously related to poor clinical outcomes and metastasis [38,39]; MCP-1, a crucial chemokine in the activation of TAMs expressed by stromal cells [40], high levels of which were observed after SW620 EV treatment; and TIMP-1, an MMP inhibitor, decreased levels of which might accentuate MMP activity of cancer cells and subsequent ECM degradation. However, increased expression of TIMP-1 in cancer cells has been also associated with cancer progression [41]. Extravesicular TIMP-1 activity was extensively investigated in stromal fibroblasts, which induced TIMP-1 release by fibroblasts, as well as ECM remodelling and cancer invasion [42]. Due to the decreased levels observed after EV treatment, it was of interest to investigate TIMP-1 and lesser known TIMP-2 among the targets—with TIMP-2 contributing a higher VIP score in the multivariate analysis.

EVs of different sizes were observed after processing plasma through SEC. Plasma EVs are commonly found with tetraspanin percentages lower than 10% with this technique [43], as an indication of the degree impurity (albumin and lipoproteins) typical of EV isolation from blood. This was confirmed by the marker analysis performed with nanoFCM, which showed lower percentages of all three tetraspanins. When the markers were examined by DELFIA, a high-sensitivity immunoassay, the presence of CD9, CD63, and CD81 was observed in SEC fractions 8–12 and they were absent in larger particle fractions. The DELFIA immunoassay is commonly used for the detection of target antibodies in blood [44]. With DELFIA, it was also possible to identify CD9, CD63, and CD81 directly in plasma, without the necessity of EV isolation. These results demonstrated the potential of DELFIA in the exploration of EV markers in blood. Alongside the presence of protein and lipoprotein contaminants, EVs derived from liquid biopsies can have different origins, and thus variations in marker expression cannot be exclusively linked to the presence of a disease condition [45], which adds to the limitation of detecting specific markers. This preliminary study aimed to establish the relevance of cellular responses to patient-derived EVs in the discrimination of the stage of the disease.

When observed individually, only some of the cell activities and markers analysed were affected by the plasma EV stage. However, none of the variations were statistically significant when compared between the three patient groups. Higher expression of GDF-15 and VEGF was observed after treatment with M1 EVs compared to HC EVs. In both cell markers, statistical significance would have been reached if not for one outlying value. More variable were the changes upon treatment with early stage M0 EVs, which often were comparable to those upon treatment with HC EVs.

When validating potential biomarkers, the selection of patients plays a crucial role in data interpretation. For this pilot study, healthy controls were recruited to match as close as possible the characteristics of the CRC patient cohort. However, it was not possible to completely match both age and sex. Furthermore, since outlying values from the cluster of the late-stage group were observed, patient history and information were considered. In VEGF, GDF-15, and MCP-1, the patient distanced from the late-stage cluster was the only patient with stage IV CRC. The patient was also under levothyroxine medication, which is given to patients with underactive thyroid [46]. Levels of factors and homeostasis in the blood can indeed be altered by specific treatments and medications [47]. In IL-6 and IL-8, the only two diabetic patients showed very different values from the cluster. Drug treatments and co-morbidities can also have an impact in the secretion and functionality of EVs, driving different responses, which might compromise the interpretation of the functional role of the biomarkers identified [48]. Despite the preliminary nature of the study, this suggests important information about the feasibility of cellular responses to plasma EVs as cancer biomarkers and reinforces carefully selection of cohorts of patients for future work aiming to expand this current study.

With the purpose of adding power to the pilot study, all of the assay results were included in a multivariate analysis model, including the DELFIA analysis of CD9, CD63, and CD81 in the whole plasma. In the PCA, partial separation was observed, driven by outliers from M1 and HC parting from the main cluster. In combination with PCA, supervised tests such as PLS-DA are also performed to classify samples and identify important variables by introducing information about the sample groups [49]. When PLS-DA was performed, separation between the groups was more evident, with complete separation between M0 and M1. From the VIP scores, it was concluded that a combination of EV markers tested in plasma and cellular responses to plasma EVs was responsible for the separation in a stage-dependent manner. Multivariate analysis gains power with larger amounts of data and the cohort of patient selected in this pilot work was too small to give robust information about the novel biomarkers. Thus, increasing the patient number in the cohort would contribute to gaining significance; indeed, our power analysis showed the estimated number of patients per group needed to validate the findings should be between 60 and 100 for a full follow-up clinical study.

In conclusion, the use of cellular responses, as multiplexed bioassays, demonstrates that cancer EVs can determine differential activities in cells according to the disease stage. Despite the complex nature of blood components in liquid biopsies, stage-related effects were also partially observed ex vivo with plasma EVs. However, the functional effects failed to consistently mirror the effects derived from in vitro treatments. Preliminary supervised multivariate analysis showed promising results for using the model based on cellular responses to EVs as a screening test. Moreover, it showed EV-mediated GDF-15 release by cancer cells worthy of follow-up study. The results obtained were limited in the number of patients and therefore need to be considered as preliminary. In future, it is pivotal to expand the study with larger numbers of patients in different stages of disease, also accounting for medications and confounding factors such as age, sex, and co-morbidities that could influence the outcome. This work provides a new perspective in the investigation of EVs as cancer biomarkers and the development of screening technologies to support cancer diagnosis and prognosis.

## 4. Materials and Methods

### 4.1. Cell Culture

The primary adenocarcinoma SW480 cell line and patient-matched secondary adenocarcinoma SW620 cell line were purchased from the European Collection of Authenticated Cell Cultures (ECACC), while human lung fibroblast cell line (MRC5) was purchased from Sigma-Aldrich^®^ (Gillingham, UK). All cells were cultured in complete Dulbecco’s Modified Eagle Medium (cDMEM), which was comprised of high glucose DMEM + GlutaMAX + pyruvate (Gibco, Thermo-Fisher, Cramlington, UK), 10% *v*/*v* foetal bovine serum (FBS; Gibco, Thermo-Fisher, Cramlington, UK), and 1% *v*/*v* penicillin/streptomycin (P/S; Lonza Ltd., Slough, UK). The cells were maintained in a humidified incubator at 37 °C and 5% CO_2_ in air. The culture medium was replaced every 3–4 days and cells sub-cultured (ratios 1:10 SW480/SW620, 1:5 HFF2/MRC5) with Trypsin-EDTA (Thermo-Fisher, Loughborough, UK) when reaching 70–80% confluence. Cells were checked for mycoplasma using the MycoAlert© Detection kit (Lonza Ltd., Slough, UK) every 6 months and were shown to be negative throughout.

### 4.2. Isolation of SW480 and SW620 EVs

SW480 or SW620 cells were cultured in WHEATON^®^ CELLine™ AD-1000 Bioreactor flasks (DWK Life Sciences, GmbH, Wertheim, Germany). Briefly, cells (2.5 × 10^7^) in 15 mL cDMEM were added to the inner cell compartment. cDMEM (500 mL) was then added to the outer medium compartment. The cells were left to grow and adhere to the membrane for 10 days. Then, the medium in the cell compartment was replaced after washes in phosphate-buffered saline (PBS) with DMEM supplemented with 10% *v*/*v* Gibco EV-depleted FBS (Thermo-Fisher, Cramlington, UK). The medium in the outer compartment was replaced with 5% normal FBS and 1% P/S in DMEM. The medium in the outer compartment was replaced weekly. The EV-enriched conditioning medium (CM), harvested weekly, was centrifuged at 300× *g* for 5 min (21 °C). The supernatant was collected and centrifuged again at 2000× *g* for 5 min (21 °C). The supernatant was then used to isolate EVs on the same day or stored at −80 °C until further use.

The CM was concentrated to reach a volume of 0.5 mL and ultrafiltered through Vivaspin^®^ 20 (100 kDa MWCO) (Sartorius, Goettingen, Germany) at 3000× *g* for 4 °C. EVs were separated from soluble factors by SEC, loaded on Econo-Pac columns (Biorad, Watford, UK) with 10 mL sepharose CL-2B (GE Healthcare, Uppsala, Sweden), and eluted in PBS. Twenty fractions of 0.5 mL were obtained from each column.

### 4.3. Isolation of Plasma EVs

Blood from CRC patients was collected in EDTA vacutainers after obtaining written informed consent following ethical approval (REC reference: 19/NI/0221) from the Department of General Surgery, Sheffield Teaching Hospitals. Similarly, healthy control blood from volunteers at Sheffield Hallam University was collected after obtaining written informed consent (Table 1). For this pilot study, 6 patients per group were chosen, representing different stages of the disease: 6 patients with CRC but no evidence of nodal or metastatic involvement (N = 0, M = 0), and 6 patients with either lymphatic spread (N ≠ 0) or distant metastasis (M ≠ 0). Within three hours of collection, the blood was centrifuged at 1000× *g* for 10 min to obtain whole plasma. The plasma was diluted 1:1 and centrifuged at 1500× *g* for 15 min. Again, the supernatant was centrifuged at 2500× *g* for 15 min before storing it at −80 °C. EVs were isolated within 6 months of storage. Once defrosted, 0.5 mL of plasma was centrifuged to remove the lipoproteins at 10,000× *g* for 30 min and then applied to an SEC column, as described above.

### 4.4. Protein Quantification with BCA Assay

Protein quantification assay was performed on SEC fractions 1–20 using the Pierce™ bicinchoninic acid (BCA) assay kit (Thermo Fisher, Cramlington, UK). Briefly, bovine serum albumin (BSA) was used to generate 1:2 serial dilutions between 15.625 and 2000 µg/mL as a standard curve. Then, 10 µL aliquots of samples and standard were placed in a 96-well plate in duplicate, with 100 µL of working reagent, consisting of copper sulphate II solution (Sigma-Aldrich^®^, Gillingham, UK) and BCA solution (Sigma-Aldrich^®^, Gillingham, UK) in a 1:50 ratio. The plate was incubated at 37 °C for 1 h and then the absorbance was read at 570 nm.

### 4.5. Transfer Electron Microscopy

Transfer electron microscopy (TEM) of EVs was performed at the Electron Microscopy Facility, Faculty of Science, University of Sheffield. EVs were transferred through absorption onto a carbon-coated copper grid for 1 min, quickly dried with filter paper, and then washed twice with water. Then, filtered 2% uranyl acetate was used for 2 min to negatively stain the EVs. Excess liquid was removed with filter paper, the grid was allowed to dry for 10 min, and the grid was then stained with uranyl formate. Grids were visualised on a FEI Tecani G2 Spirit BioTwin (PennState, State College, PA, USA) TEM, and images were recorded using a Gatan Orius 1000B CCD camera and Gatan Digital Micrograph software (v. 3.5, Gatan, Pleasanton, CA, USA) at the University of Sheffield Facility.

### 4.6. NanoFCM

Pooled EVs were characterised for size, particle count, and tetraspanin markers (CD9, CD81, CD63) using a nanoanalyser instrument based on nanoflow cytometry (nanoFCM) through collaboration with NanoFCM Co., Ltd. (MediCity, Nottingham, UK). Briefly, 10 μL of unlabelled EVs was first analysed to assess particle count and size. EVs were compared to QC beads (250 nm silica standard) and size beads (68, 91, 113, and 155). Then, 2 × 10^8^ particles were labelled with FITC/APC conjugated antibodies for CD9, CD81, and CD63 (1:10) and incubated at RT for 30 min. Finally, 10 μL was run on the instrument.

### 4.7. Western Blotting for TSG101 and GM130

To confirm the presence of EVs in the SEC fractions, positive (TSG101) and negative (GM130) markers of EVs were investigated by western blotting. Cell lysates were used as negative controls to evaluate marker specificity. Antibodies against TSG101 (ab2386 mouse monoclonal [CUB 7402]; Abcam, UK; 1:1000 dilution) and Golgi matrix protein GM130 (ab52649 rabbit monoclonal [EP892Y]; Abcam, UK; 1:1000 dilution) were used. A volume of 15 µL of determined SEC fractions was loaded in 10% sodium dodecyl sulphate (SDS)-polyacrylamide resolving gels and electrophoresed in running buffer (0.25 M Tris, 1.92 M glycine, 1% *w*/*v* SDS) at 120 V for 1.5 h. The resolved proteins were transferred onto the nitrocellulose membrane in transfer buffer (1:1:5 RTA Transfer Kit: EtOH:dH_2_O) (Bio-Rad, Watford, UK) using the Trans-Blot^®^ Turbo™ transfer system (Bio-Rad, Watford, UK) for 7 min. The membrane was then blocked with blocking buffer (5% *w*/*v* non-fat dried milk (Marvel, Dublin, Ireland) with 0.05% Tween20 washing buffer in Tris-buffered saline) at RT for 1 h. TSG101 and GM130 primary antibodies were added to the blocking solution and left ON at 4 °C. The membrane was rinsed and washed three times for 10 min each with washing buffer before IRDye 800RD goat anti-mouse or IRDye 680RD donkey anti-rabbit secondary antibodies (1:10,000 dilution; LI-COR Biosciences, Cambridge, UK) were added, followed by incubation at RT for 1 h. The membrane was washed again three times in washing buffer and then analysed using the Li-Cor^®^ Odyssey^®^ instrument (LI-COR Biosciences, Cambridge, UK).

### 4.8. DELFIA Immunoassay

To confirm tetraspanin expression, blood EVs and pre-SEC plasma were tested for CD9 (ab2215 mouse monoclonal [MEM-6]; Abcam, Cambridge, UK; 1:5000 dilution), CD63 (MCA2142 mouse monoclonal [MEM-259]; Bio-Rad, Watford, UK; 1:5000 dilution), and CD81 (MCA1847 mouse monoclonal [1D6]; Bio-Rad Ltd., Watford, UK; 1:5000 dilution) with dissociation-enhanced lanthanide fluorescence immunoassay (DELFIA©). SEC fractions 5–12 were incubated ON at 4 °C in a Nunc 96-well MaxSorp™ ELISA plate. Between every incubation step, the plate was washed four times with 200 µL/well of washing buffer (10 mM Tris-HCl, 0.05% *v*/*v* Tween-20, 130 mM NaCl) and blotted with a clean paper towel to remove all of the buffer. Blocking of non-specific sites was performed by adding 1% *w*/*v* BSA in PBS for 2 h at RT. Primary antibodies for CD9, CD63, and CD81 (0.1% *w*/*v* BSA in PBS) were incubated ON at 4 °C. The plate was then incubated with biotinylated anti-mouse IgG secondary antibody (1:2500, Perkin-Elmer Life Sciences, Beaconsfield, UK) in 0.1% *w*/*v* BSA in PBS for 1 h at RT onto a plate shaker. Eu-N1 streptavidin conjugate (Perkin-Elmer Life Sciences, Beaconsfield, UK) was diluted (1:1000) in assay buffer (50 mM Tris-HCl, 0.9% *v*/*w* NaCl, 0.2% *v*/*w* BCA, 0.1% *v*/*v* Tween-20, 20 µM pentetic acid) and added to the wells for 45 min at RT on a plate shaker. The plate was washed six times in washing buffer before the last incubation in DELFIA© enhancement solution (Perkin-Elmer Life Sciences, Beaconsfield, UK) for 5 min at RT on a plate shaker. TRF was measured using the Europium protocol (ex/em 320/615 nm) with a Wallac Victor2™ 1420 multilabel counter (Perkin-Elmer Life Sciences, Beaconsfield, UK).

### 4.9. Alamar Blue Assay

SW480 or MRC5 cells (20,000 cells/well) were cultured in a 96-well plate and left growing until reaching 50% confluence. Then, cells were treated with 50 μg/mL of SW480/SW620 EVs or 10 µL plasma EVs in DMEM (+10% EV-dep serum for 48 h). A control condition (PBS) was also created for each experimental replicate. The supernatant was collected from the wells at the end of the experiment and stored at −80 °C for further assays. Resazurin sodium salt was resuspended in EV-dep media to reach a concentration of 0.03 mg/mL and used to replace the CM of the cultures for 4 h. Finally, absorbance readings were taken with an excitation wavelength (λex) at 530–560 nm and emission wavelength (λem) at 590 nm using a Clariostar instrument (BMG Labtech, Aylesbury, UK). For assays of chemotoxicity, cells were treated with 10 µM oxaliplatin in combination with EVs, and the plate was re-read after 72 h before normalisation to pre-treatment readings.

### 4.10. Scratch Assay

SW480 or MRC5 cells (500,000 cells/well) were cultured in a 24-well plate and left growing until reaching 100% confluence. Then, a pipette tip was used to create a vertical scratch of about 1 mm in the middle of the culture. After three PBS washes to remove the detached cells, adherent cells were treated with 50 μg/mL of SW480 EVs/SW620 EVs or 50 µL of plasma EVs in DMEM + 10% EV-dep serum. A control condition (PBS) was also created for each experimental replicate. Brightfield images were acquired with the Cytation™ 5 Cell Imaging Multi-Mode Reader (BioTek, Agilent, Cheadle, UK) at 0, 8, 24, and 36 h of culture. The supernatant was collected from the wells at the end of the experiment and stored at −80 °C for further assays. Images of the scratch time points were analysed with ImageJ 5.1i. Ten measurements of the distance in the scratch gap were taken at each time point and the difference was calculated to evaluate the migration length of the cells.

### 4.11. Glucose Uptake

Glucose consumption in the conditioned supernatant was assessed through the glucose oxidase assay (Gox). Briefly, glucose oxidase reagent was prepared by adding 1 mg/mL of 2,2′-azino-bis (3-ethylbenz-thiazoline-6-sulfonic acid) disodium salt (ABTS), 1000 µL/mg of peroxidase type VI-A, and 40.3 µL/mg of glucose oxidase type II-S to 0.1 M phosphate-buffered solution (pH 7). A standard curve of glucose (0–100 μM) was generated and 50 μL of each standard or sample was added to a 96 well-plate. Glucose oxidase reagent was added to the solution and the colorimetric optical density (OD) was measured at 570 nm.

### 4.12. Lactic Acid Production

Lactic acid production was measured using the L-lactic acid (L-Lactate) assay kit (Megazyme, Bray, Ireland) as per the manufacturer’s instructions. A standard curve was prepared with L-lactic acid (0–15 μg/mL). Standards and samples were mixed with solution buffer (pH 10.0) plus D-glutamate and sodium azide (0.02% *w*/*v*), NAD+/PVP, and D-GPT. Then, L-LDH was introduced and the mixture was incubated for 10 min before the OD was measured at 340 nm.

### 4.13. Cytokine Arrays

Cytokine arrays were employed for the discovery of differential expression of cytokines upon EV treatment. Human cytokine arrays (ab133998 and ab169815, Abcam, UK) were performed according to the manufacturer’s protocol, testing 80 targets for MRC5 cells and 51 targets for SW480 cells. Membranes were initially blocked with blocking buffer (1×) at RT for 30 min. The buffer was replaced by 1 mL of supernatant from the scratch assay (PBS control and SW620 EV treatment) and left at 4 °C ON. For each incubation step, 6 washes (5 min each, RT) were performed with two different washing buffers. After sample incubation, a large volume wash was also performed to remove the excess (45 min). The membranes were incubated ON at 4 °C with biotin-conjugated anti-cytokines. Then, washing steps were performed and HRP-conjugated streptavidin was added to the membrane for 2 h at RT. To detect signals, detection buffers were mixed 1:1 and added to the membrane for 2 min before chemiluminescent images were obtained using the LI-COR OdysseyFc Imager (LI-COR Biosciences, Cambridge, UK). Semi-quantitative densitometry analysis was performed with Image Studio Lite (LI-COR Biosciences, Cambridge, UK).

To choose relevant cytokines for further analysis, density values and fold change compared to PBS treatment were considered. Cytokines with density values under 0.01 were discarded. Fold changes with opposing results between biological replicates were discarded.

### 4.14. Cytokine Release (ELISA)

All of the ELISA assays were performed according to the manufacturer’s instructions for commercially available kits (PeproTech EC Ltd., London, UK; Bio-Techne, Abingdon, UK). Capture antibody, detection antibody, and streptavidin-HRP concentrations alongside the supernatant dilutions are reported in Table 2, as well as the ELISA assay antibody concentrations and standard curve ranges. For VEGF-165, concentrations were approximately calculated as information was not provided by the supplier. Nunc 96-well MaxSorp™ ELISA plates (Thermofisher, Cramlington, UK) were coated with 50 µL/well of capture antibodies and incubated ON at RT. Between every incubation step, the plates were washed four times with 200 µL/well of washing buffer (0.05% Tween-20 in PBS) and blotted with clean paper towel to remove all of the buffer. The following morning, the plates were incubated with 200 µL/well of blocking buffer (1% BSA in PBS) for 1 h at RT. Then, the plates were incubated with 50 µL/well of samples/standards ON at 4 °C. Dilution antibodies (50 µL/well) were incubated for 2 h at RT, followed by 30 min incubation at RT with 50 µL/well of streptavidin. The final incubation step was performed with 50 µL/well of TMB substrate (Fisher Scientifics, Loughborough, UK). Without washing the plates, the reaction was stopped by adding 50 µL/well of 1 M HCl. Ultimately, the optical density of the reaction was analysed using a Clariostar plate reader at a 450 nm wavelength, with the correction set at a 620 nm wavelength.

### 4.15. Statistical Analysis

Statistical analysis was performed using Prism 8.1.1 software (Dotmatics, Boston, MA, USA). First, data were tested for normality (Gaussian distribution) with two modalities: D’Agostino and Pearson test (for data with n > 3) and Shapiro–Wilk test (for data with n ≤ 3).

As the groups tested (CRC EV and plasma EV treatments) were ≥3, multiple comparison tests were employed for all datasets. Multiple comparison through ordinary one-way ANOVA (ANOVA) with Tukey’s test as post hoc analysis was performed for parametric data, while the Kruskal–Wallis (KW) test with Dunn’s test as post hoc analysis was performed for non-parametric data. *p*-Values less than 0.05 were considered significant.

Data representation varied according to the number of replicates and distribution. For data with n ≤ 6, individual values are shown. When data are shown as a summary, mean ± SEM was used for parametric data, whereas medium ± 95% CI was used for non-parametric data.

### 4.16. Multivariate and Power Analyses

Multivariate analysis from the cellular responses assays tested on SW480 and MRC5 cells after plasma EV conditioning was performed with opensource Metaboanalyst (v. 5.0, Xia Lab, https://www.metaboanalyst.ca/MetaboAnalyst/home.xhtml, accessed on 12 October 2023). Normalisation by sum and autoscaling were performed as pre-processing. Unsupervised PCA and supervised PLS-DA were performed. From the PLS-DA, VIP scores were obtained. Finally, a heatmap for the three groups (HC, M0, and M1) was obtained.

Power analysis was performed with Metaboanalyst with the same parameters applied for the multivariate study, normalisation by sum and autoscaling applied as pre-processing. The main comparisons were performed between M0 and M1. The predicted power curve was for FDR = 0.1 (significance criterion) and a maximum size cohort of 100 patients per group.

## Figures and Tables

**Table 1:** List of patients selected for the blind study. Blood was collected within the period of 6 months. * Age and symptoms of CRC patients and healthy donors at sample collection. CRC patients divided into two groups according to their tumour, node, and metastasis (TNM) stage. M0 = T2–3; M1 = T3–4.

Sex	Age *	Classification	Symptom Duration *	Medication Details
F	57	/	/	/
F	39	/	/	/
F	49	/	/	/
F	45	/	/	/
M	44	/	/	/
F	43	/	/	/
F	85	T2N0M0	3 months	Atenolol, atorvastatin, clopidogrel, lansoprazole
M	83	T2N0M0	0 months	Bisoprolol, isosorbide mononitrate, rampiril, aspirin, ezetimibe
F	78	T2N0M0	2 months	Ferrous fumarate, pregabalin, amiloride, amitriptyline, apixaban, bisoprolol, domperidone, esomeprazole, furosemide, quinine
M	89	T2N1M0	4 months	Tamsulosin, tolteradine, allopurinol, amlodopine, atorvastatin, omperpazole, solifenacin
M	83	T3N0M0	0 months	Lansoprazole, simvastatin
M	85	T3N0M0	12 months	Felodopine, alogliptin
M	85	T3N0M1	2 months	Ursedoxycholic acid, calcium carbonate, alendronic acid
M	80	T3N1M1	0 months	Insulin, rivaroxaban, atorvastatin, bisoprolol, clopidogrel, furosemide, isosorbide mononitrate, lansoprazole, ramipril, ranolazine, colecalciferol
M	64	T3N1M1	0 months	Ramipril, doxyxyline, lansoprazole
M	63	T3N2M1	1 month	Adalumimab
F	72	T4N0M1	9 months	/
M	78	T4N2M1	1 month	Finasteride, carcbocisteine, omeprazole, colecalciferol, omeprazole, atenolol, felodopine, simvastatin

**Table 2:** ELISA assay antibody concentrations and standard curve ranges. For VEGF-165, concentrations were approximately calculated as information was not provided by the supplier.

Cytokine	Capture Ab (µg/mL)	Detection Ab (µg/mL)	Standard Curve (pg/mL)
IL-6 900-T16 (Peprotech, London, UK)	0.5	0.125	7.81–2000
IL-8 900-T18 (Peprotech, London, UK)	0.125	0.25	0.78–200
MCP-1 DY279 (Bio-Techne, Abingdon, UK)	1	0.025	15.6–1000
TIMP-1 DY970 (Bio-Techne, Abingdon, UK)	2	0.05	31.2–2000
TIMP-2 DY971 (Bio-Techne, Abingdon, UK)	2	0.0125	31.2–2000
GDF-15 DY957 (Bio-Techne, Abingdon, UK)	2	0.0125	7.81–500
VEGF-165 900-T10 (Peprotech, London, UK)	≈0.1	≈0.1	3.125–800
Nidogen-1 RK07569 (Universal biologicals, Cambridge, UK)	0.5	0.125	0.075–10,000

## Data Availability

Raw data files are stored on a secure server under institutional data retention policies. The corresponding author can provide fully anonymised raw data upon request.

## Supplementary Materials

The following supporting information can be downloaded at: https://www.mdpi.com/article/10.3390/ijms242316755/s1.
