# Supplementary material for: Cellular Responses to Extracellular Vesicles as Potential Markers of Colorectal Cancer Progression
Source: Int J Mol Sci. 2023 Nov 25;24(23):16755. doi: 10.3390/ijms242316755 (PMC10706375; doi:10.3390/ijms242316755)
Supplement: Supplementary file 1 [file ijms-24-16755-s001.zip › ijms-2688902-supplementary.pdf]

International Journal of
Molecular Sciences

Supplementary Materials

Figure S1. SW480 and MRC5 cell migration upon treatment with SW480/SW620 EVs

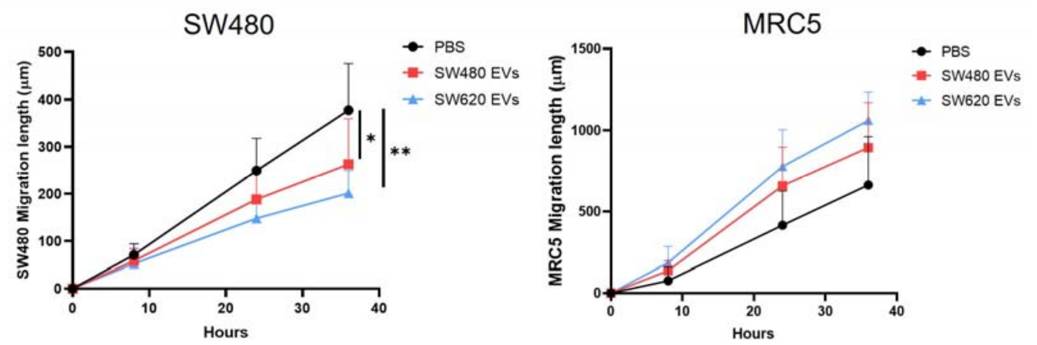

Figure S1. Scratch time points for SW480 and MRC5 migration. Scratch assay was performed at 0, 8, 24, 36 hours for SW480 and MRC5 after treatment with SW480 and SW620 EVs. One-way ANOVA with Tukey's post hoc test, *p < 0.05, **p < 0.01.

Table S1. Cytokine array on SW480 supernatant.

	CTRL		SW620		Fold change	
	R1	R2	R1	R2	R1	R2
Adiposin	0.02235	0.01255	0.029452	0.009858	1.3177	0.7854
BCAM	0.0359	0.01465	0.048402	0.015765	1.3482	1.0760
CD30	0.01625	0.00818	0.025596	0.006166	1.5751	0.7537
CD40	0.01105	0.00688	0.018229	0.005707	1.6493	0.8288
Fcr RIIB/C	0.0121	0.00607	0.017958	0.006119	1.4841	1.0072
Ferritin	0.06415	0.0769	0.064236	0.051543	1.0013	0.6702
FLRG	0.02005	0.01325	0.02092	0.008877	1.0433	0.6699
Follistatin	0.03315	0.0225	0.042249	0.017082	1.2744	0.7592
Furin	0.02215	0.00942	0.012626	0.009391	0.5700	0.9963
Galectin-7	0.00626	0.00367	0.00594	0.003255	0.9480	0.8869
GDF-15	0.226	0.13	0.559494	0.222234	2.475	1.7094
Growth Hormone	0.0195	0.01004	0.026826	0.010241	1.3756	1.0194
IL-10 R α	0.0289	0.01405	0.042332	0.014787	1.4647	1.0524
IL-22	0.0187	0.00929	0.025924	0.009688	1.3863	1.0428
IL-28A	0.00946	0.00522	0.020264	0.005099	2.1420	0.9758
IL-29	0.0277	0.0133	0.049715	0.015042	1.7947	1.1309
IL-31	0.01305	0.00827	0.020756	0.007491	1.5904	0.9058
Insulin	0.0086	0.00440	0.014061	0.004967	1.635	1.1276
Luteinizing Hormone	0.0194	0.01012	0.02133	0.011261	1.0994	1.1126
LIMPII	0.00671	0.00338	0.006465	0.009255	0.9634	2.7381
LYVE-1	0.0158	0.00859	0.007786	0.00583	0.4927	0.6786
Maraspin	0.0106	0.00589	0.013077	0.004742	1.2336	0.8050
MICA	0.03185	0.017	0.037983	0.018144	1.1925	1.0673

MICB	0	0	0.000281	0	0	0
MMP-2	0.0155	0.00774	0.025842	0.008923	1.6672	1.1528
MMP-7	0.014	0.00711	0.033389	0.007377	2.3849	1.0368
MMP-8	0.0123	0.00565	0.023299	0.00546	1.8942	0.9664
MMP-10	0.0268	0.01265	0.050864	0.013258	1.8978	1.0480
NCAM-1	0.046	0.01735	0.0781	0.024178	1.6978	1.3935
Nidogen-1	0.1495	0.0815	0.379012	0.10878	2.5351	1.3347
NrCAM	0.0305	0.01535	0.038065	0.013555	1.2480	0.8830
NRG1-β1	0.02385	0.01525	0.02699	0.013725	1.1316	0.9
Osteopontin	0.01625	0.00947	0.007679	0.007173	0.4725	0.7570
PAI-I	0.232	0.1485	0.255136	0.161895	1.0997	1.0901
Platelet Factor 4	0.01785	0.00811	0.02658	0.007678	1.4890	0.9461
PSA-total	0.01245	0.00645	0.017966	0.004547	1.4430	0.7049
RAGE	0.01115	0.00530	0.014972	0.004781	1.3427	0.9011
RANK	0.00649	0.00270	0.009664	0.003837	1.4879	1.4184
Resistin	0.00062	0	0	0.000134	0	1.5845
SAA	0.01875	0.00856	0.029288	0.007292	1.562	0.8513
Siglec-9	0.019	0.00936	0.037491	0.006777	1.9732	0.7237
TACE	0.0241	0.01185	0.035194	0.011218	1.4603	0.9466
TIM-1	0.01155	0.00595	0.014767	0.006646	1.2785	1.1160
TRAIL R2	0.0182	0.00985	0.023135	0.010071	1.2711	1.0223
Trappin-2	0.0106	0.00915	0.00617	0.006697	0.5820	0.7314
TREM-1	0.0145	0.00906	0.02174	0.008108	1.49931	0.8948
TSH	0.02905	0.01455	0.041921	0.017167	1.44306	1.1798
TSLP	0.0123	0.00695	0.012199	0.006913	0.9917	0.9940
VCAM-1	0.01255	0.00717	0.011239	0.006183	0.89553	0.8622
VEGF-C	0.01465	0.00931	0.012167	0.007513	0.8304	0.8069
XEADR	0.02665	0.01415	0.023299	0.01092	0.8742	0.7717

Table S2. Cytokine array on MRC5 supernatant.

	CTRL		SW620		Fold change	
	R1	R2	R1	R2	R1	R2
ENA-78	0.101	0.0182	0.110055	0.029407	1.0896	1.6158
GCSF	0.0107	0.00169	0.007956	0.006023	0.7435	3.5639
GM-CSF	0.0065	0	0.005828	0	0.8857	0
GRO	0.109	0.0114	0.088177	0.013741	0.8100	1.2053
GRO-α	0.0573	0.0143	0.050785	0.010222	0.8863	0.7148
I-309	0.0318	0.00645	0.022674	0.006254	0.7130	0.9696
IL-1α	0.0383	0.00203	0.030166	0.00967	0.7876	4.7635
IL-1β	0.0727	0.00755	0.0661	0.022216	0.9092	2.9425
IL-2	0.0161	0.00127	0.012928	0.009066	0.8029	7.1385
IL-3	0.0677	0.00488	0.088177	0.031462	1.3024	6.4471
IL-4	0.0266	0.00301	0.025923	0.007949	0.9745	2.6408
IL-5	0.0229	0	0.019558	0.003942	0.8540	0
IL-6	0.957	0.0198	1.200002	0.030435	1.2539	1.5371
IL-7	0.00391	0	0.002446	0.001913	0	0

IL-8	0.568	0.0925	0.5642	0.25298	0.9933	2.7349
IL-10	0.0571	0.00944	0.0541	0.004867	0.9474	0.5155
IL-12	0.0617	0.0278	0.059403	0.014254	0.9627	0.5127
IL-13	0.0478	0	0.047536	0.000119	0.9944	0
IL-15	0.0426	0.00787	0.050387	0.018749	1.1827	2.3823
IFN-γ	0.0666	0.011	0.078895	0.027738	1.1846	2.5216
MCP-1	1.22	0.213	1.538124	0.602272	1.2607	2.8275
MCP-2	0.0258	0.00504	0.030961	0.011069	1.2000	2.1962
MCP-3	0.0324	0.00613	0.041304	0.006883	1.2748	1.1228
MCSF	0.0955	0.0164	0.104752	0.03159	1.0968	1.9262
MDC	0.0583	0.00999	0.069613	0.020161	1.1940	2.0181
MIG	0.0811	0.00461	0.052243	0.023115	0.6441	5.0140
MIP-1b	0.141	0.0353	0.131934	0.015282	0.9357	0.4329
MIP-1δ	0.046	0.00665	0.033083	0.010196	0.7191	1.5332
RANTES	0.104	0.0115	0.096133	0.023243	0.9243	2.0211
SCF	0.0534	0.00811	0.043624	0.018492	0.8169	2.2801
SDF-1	0.0574	0.0115	0.045216	0.068317	0.7877	5.9406
TARC	0.0491	0.00593	0.067624	0.015667	1.3772	2.6419
TGF-β1	0.0115	0.00074	0.017768	0.013741	1.5450	18.4690
TNF-α	0.0585	0.00862	0.053635	0.017978	0.9168	2.0856
TNF-β	0.0401	0.00673	0.033348	0.014254	0.8316	2.1179
EGF	0.0888	0.015	0.092155	0.029407	1.0377	1.9604
IGF-1	0.218	0.0278	0.129945	0.060356	0.5960	2.1710
Angiogenin	0.438	0.0596	0.424973	0.053036	0.9702	0.8898
Oncostatin M	0.143	0.0216	0.116685	0.037112	0.8159	1.7181
Thrombopoietin	0.0578	0.0087	0.034608	0.014254	0.5987	1.6383
VEGF	0.0827	0.0128	0.066961	0.024784	0.8096	1.9362
PDGF-BB	0.0451	0.00753	0.040972	0.014254	0.9084	1.8929
Leptin	0.0824	0.0074	0.076243	0.017721	0.9252	2.3947
BDNF	0.507	0.0526	0.716023	0.086553	1.4122	1.6454
BLC	0.0456	0.0078	0.03368	0.018364	0.7385	2.3543
Ck β 8-1	0.0822	0.0129	0.074254	0.024528	0.9033	1.9013
Eotaxin	0.0552	0.0185	0.065039	0.022344	1.1782	1.2077
Eotaxin-2	0.0641	0.0341	0.048066	0.017336	0.7498	0.5083
Eotaxin-3	0.0385	0.0117	0.033348	0.010338	0.8661	0.8835
FGF-4	0.0543	0.0115	0.042961	0.018235	0.7911	1.5856
FGF-6	0.0849	0.0179	0.057547	0.023243	0.6778	1.2984
FGF-7	0.0631	0.00335	0.043492	0.009451	0.6892	2.8211
FGF-9	0.102	0.0208	0.086851	0.037112	0.8514	1.7842
Flt-3 Ligand	0.0585	0.0106	0.044221	0.011776	0.7559	1.1109
Fractalkine	0.0476	0.0105	0.046541	0.022601	0.9777	2.1524

GCP-2	0.0573	0.0127	0.050718	0.019391	0.8851	1.5268
GDNF	0.0928	0.0149	0.093481	0.025812	1.0073	1.7323
HGF	1.95	0.401	1.809947	0.395522	0.9281	0.9863
IGFBP-1	0.0752	0.0105	0.055823	0.015153	0.7423	1.4431
IGFBP-2	0.131	0.0261	0.082873	0.029022	0.6326	1.1119
IGFBP-3	0.0745	0.0109	0.066961	0.012649	0.8988	1.1604
IGFBP-4	0.0843	0.0109	0.071602	0.009015	0.8493	0.8270
IL-16	0.0964	0.0155	0.071602	0.01618	0.7427	1.0438
IP-10	0.118	0.0244	0.099448	0.01862	0.8427	0.7631
LIF	0.143	0.02	0.099448	0.017336	0.6954	0.8668
LIGHT	0.0773	0.0216	0.065702	0.020547	0.8499	0.9512
MCP-4	0.0404	0.00806	0.036995	0.007641	0.9157	0.9480
MIF	0.0714	0.0139	0.055293	0.013355	0.7744	0.9607
MIP-3α	0.0612	0.0119	0.019558	0.010736	0.3195	0.9021
NAP-2	0.192	0.014	0.081547	0.011994	0.4247	0.8567
NT-3	0.156	0.0135	0.07558	0.013741	0.4844	1.0178
NT-4	0.0849	0.00972	0.066298	0.008578	0.7808	0.8825
Osteopontin	0.132	0.0168	0.08884	0.007243	0.6730	0.4311
Osteoprotegerin	2.74	0.593	2.333705	0.410932	0.8517	0.6929
PARC	0.0929	0.0112	0.06358	0.005252	0.6843	0.4689
PIGF	0.075	0.00888	0.051514	0.006151	0.6868	0.6926
TGF-β2	0.0448	0.00112	0.028044	0.001926	0.6259	1.7196
TGF-β3	0.0856	0.0108	0.056884	0.006036	0.6645	0.5588
TIMP-1	1.69	0.559	1.206631	0.308199	0.7139	0.5513
TIMP-2	2.5	0.00737	1.770168	0.007975	0.7080	1.0820
